# Electroacupuncture analgesia is associated with increased serum brain-derived neurotrophic factor in chronic tension-type headache: a randomized, sham controlled, crossover trial

**DOI:** 10.1186/s12906-015-0664-x

**Published:** 2015-05-07

**Authors:** Mônica Chassot, Jairo Alberto Dussan-Sarria, Francislea Cristina Sehn, Alícia Deitos, Andressa de Souza, Rafael Vercelino, Iraci LS Torres, Felipe Fregni, Wolnei Caumo

**Affiliations:** Post-Graduate Program in Medical Sciences, School of Medicine, Universidade Federal do Rio Grande do Sul (UFRGS), Porto Alegre, Brazil; Pain and Palliative Care Service at Hospital de Clínicas de Porto Alegre (HCPA), UFRGS, Porto Alegre, Brazil; Laboratory of Pain and Neuromodulation, Hospital de Clínicas de Porto Alegre at UFRGS, Rua Ramiro Barcelos, 2350 - CEP 90035-003 Bairro Rio Branco, Porto, Brazil; Post-Graduate Program in Health and Human Development, Unilasalle Universitary Center, Canoas, Brazil; Pharmacology Department, Instituto de Ciências Básicas da Saúde, UFRGS, Porto Alegre, Brazil; Spaulding Center of Neuromodulation, Department of Physical Medicine and Rehabilitation, Harvard Medical School, Boston, Massachusetts USA; Surgery Department, School of Medicine at UFRGS, Porto Alegre, Brazil

**Keywords:** Electroacupuncture, Brain derived neurotrophic factor, Chronic tension type headache, Neuroplasticity

## Abstract

**Background:**

Chronic tension-type headache (CTTH) is characterized by almost daily headaches and central sensitization, for which electroacupuncture (EA) might be effective. The central nervous system (CNS) plasticity can be tracked in serum using the brain-derived neurotrophic factor (BDNF), a neuroplasticity mediator. Thus, we tested the hypothesis that EA analgesia in CTTH is related to neuroplasticity indexed by serum BDNF.

**Methods:**

We enrolled females aged 18–60 years with CTTH in a randomized, blinded, placebo-controlled crossover trial, comparing ten EA sessions applied for 30 minutes (2–10 Hz, intensity by tolerance) in cervical areas twice per week vs. a sham intervention. Treatment periods were separated by two washout weeks. Pain on the 10-cm visual analog scale (VAS) and serum BDNF were assessed as primary outcomes.

**Results:**

Thirty-four subjects underwent randomization, and twenty-nine completed the protocol. EA was superior to sham to alleviate pain (VAS scores 2.38 ± 1.77 and 3.02 ± 2.49, respectively, *P* = 0.005). The VAS scores differed according to the intervention sequence, demonstrating a carryover effect (P < 0.05). Using multiple regression, serum BDNF was adjusted for the Hamilton depression rating scale (HDRS) and the VAS scores (r-squared = 0.07, standard β coefficients = −0.2 and −0.14, respectively, P < 0.001). At the end of the first intervention period, the adjusted BDNF was higher in the EA phase (29.31 ± 3.24, 27.53 ± 2.94 ng/mL, Cohen’s *d* = 0.55).

**Conclusion:**

EA analgesia is related to neuroplasticity indexed by the adjusted BDNF. EA modulation of pain and BDNF occurs according to the CNS situation at the moment of its administration, as it was related to depression and the timing of its administration.

## Background

According to the International Classification of Headache Disorders, chronic tension type headache (CTTH) is characterized by daily (or almost daily) headaches that last several hours per day [1]. Its prevalence in the general population ranges from 2-5% [2,3], leads to a negative impact on both functionality and quality of life, and increases the risk for excessive analgesic consumption [4]. As with some other chronic pain conditions, patients suffering CTTH present amplification of afferent signals [5], central sensitization phenomena and inadequate descendent inhibitory control [6]. The nervous system changes induced by chronic pain are performed by different actors, including the brain-derived neurotrophic factor (BDNF).

BDNF has been identified as a key player in the sensitization of the system, altering the excitatory/inhibitory balance in the central nervous system (CNS), and in the amplification of pain transmission modulation of the nociceptive sensory inputs in the spinal cord [7,8]. BDNF is secreted by both neurons and neuroglia [9] actively crossing the blood–brain barrier. The CNS contributes to 70-80% of the serum BDNF, allowing for reliable inference of its concentration in the CNS by serum assessments [10]. Having a relevant mediator of the CNS modulation evaluated in a serum sample may open the door for improving the understanding of the neuronal plasticity in clinical scenarios, offering insights into the neuromodulation induced by current therapies, such as electroacupuncture (EA).

Although experimental studies in both animals and humans have suggested that EA effects may go beyond analgesia alone, clinical trials and meta-analyses in CTTH are inconclusive [11,12]. However, such inconclusive evidence for a single therapeutic approach is also shared by pharmacological interventions [13,14]. Nevertheless, anatomical structures analyses have found that EA increases BDNF expression in rat cortexes and hippocampi [15], where long-term depression (LTD) is additionally induced, augmenting BDNF secretion [16]. Moreover, EA has demonstrated its ability to reorganize the somatosensory cortex in patients with neuropathic pain [17], to induce LTD of postsynaptic potentials within the substantia gelatinosa [18], and to modify postsynaptic NMDA (N-methyl-D-aspartic acid) receptor expression [19]. Thus, EA represents a technique for peripheral stimulation with a CNS-modulating effect.

Striving to provide new insights into the neurobiology of CTTH and its neuroplasticity mechanisms, we designed the present cross-over trial involving applying EA and assessing the clinical outcomes and serum BDNF. We hypothesized that EA analgesia is related to changes in neuroplasticity markers as indexed by BDNF.

## Methods

The methods and results are reported according to CONSORT guidelines. The study protocol was approved by the Research Ethics Committee at the Hospital de Clínicas de Porto Alegre (HCPA) (Institutional Review Board IRB 0000921 - application n^o^.09-259) and was performed in accordance with the Declaration of Helsinki (Resolution 196/96 of the National Health Council). The protocol was registered at clinicaltrials.gov (NCT01954277). The authors confirm that all ongoing and related trials for this drug/intervention are registered.

### Design overview, setting, and participants

All patients provided their written informed consent before enrollment into this randomized, single-blinded, placebo-controlled, crossover trial. We included 34 women, aged between 18 and 60 years old, which had a clinical diagnosis of chronic tension-type headache according to the International Headache Society (The International Classification of Headache Disorders, 3rd edition (beta version)) [1]. We excluded patients with any malignancy diagnosis, rheumatoid arthritis, habitual use of anti-inflammatory steroids, decompensated systemic diseases, HIV and pregnancy.

**Figure 1:**
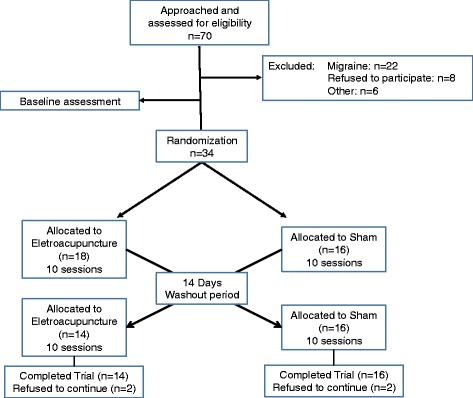
Randomization and follow-up of the study participants, CONSORT flowchart.

These subjects were recruited from a community cared by a Basic Health Unit linked to the Hospital de Clínicas de Porto Alegre (HCPA) between August 2010 and November 2012. The HCPA is a center of reference for the State of Rio Grande do Sul, receiving patients from different parts of the state. The HCPA Basic Health Unit provides health services that include social services, health prevention and promotion, as well as clinical ambulatory care, serving a population of approximately 6000 habitants. Public media advertisement (journal, radio, and television) was used to recruit patients. They were screened by an initial telephone interview, and if they met inclusion criteria, they were invited for a more in-depth encounter where medical history, and a detailed description of their headaches were determined during a 60-min structured interview. During the same encounter, the baseline biological samples were collected (see Figure 1). CTTH diagnosis was based on criteria from ***the International Classification of Headache Disorders*** (The International Classification of Headache Disorders, 3rd edition (beta version)). The participants had to experience headaches with the following characteristics for more than 180 days in the last year: pain lasting hours to days or unremitting; bilateral, pressing or tightening (non-pulsating) quality; mild or moderate intensity; and not aggravated by routine physical activities. The diagnosis was confirmed by a headache treatment specialist.

### Sample size calculation

The number of subjects in each study group was determined based on previous clinical trials [20]. An a priori estimate indicated that a total sample size of 28 patients divided into two balanced treatment arms (n = 14) was required to detect a reduction in pain intensity with EA at a minimum of 0.8 cm (average SD = 0.5 cm), with a power of 0.8 and an α-level of 0.05. (Cannella KAS, 1998). To account for the multiple outcomes and potential dropouts, we increased the sample size to 17 per group.

### Randomization

The patients were randomized into the two groups (electroacupuncture or sham). We used simple randomization to allocate the interventions, using computer software. The simple randomization relies on independent and equal probabilities to receive each intervention, for each subject. Although it is the most basic approach, it preserves unpredictability of the allocation. In our experiment, the computer software generated a random number of 5 digits for each subject, for which one of the interventions was randomly allocated. In this way, it is guaranteed that each subject had the same chance of starting the experiment receiving one or the other intervention, irrespective of the intervention allocated to the other research subjects. The envelopes contained the five digits number that represented the allocated intervention. After the participant agreed to participate in the trial and baseline assessments were performed, the opaque envelope was opened by the investigator responsible for the intervention application. After the washout period (minimum of two weeks), participants returned for completion of the second intervention period (if started with EA, participant would receive sham and vice versa). After each treatment period the baseline assessments were repeated.

### Blinding

To control for possible measurement bias in the present study, the following measures were taken: all treatment sessions were administered by the same trained (F.S.) and experienced (10 years) acupuncturist physician to ensure that the treatment was homogenous among the patients. In addition, none of the patients had undergone previous treatment with acupuncture. One evaluator who was blind to the group assignments was trained to apply the pain scales and conduct psychological tests. To avoid unblinding of the outcomes evaluators, the participants were instructed to discuss all aspects related to their treatment with the treating physician during the treatment sessions.

### Interventions

Each intervention was applied twice per week during five weeks, for a total of ten sessions. Then, a washout period of at least two weeks was granted for every participant, so they could start the second phase with the complementary intervention after collecting biological samples and performing questionnaires, again.

#### Electroacupuncture

**Figure 2:**
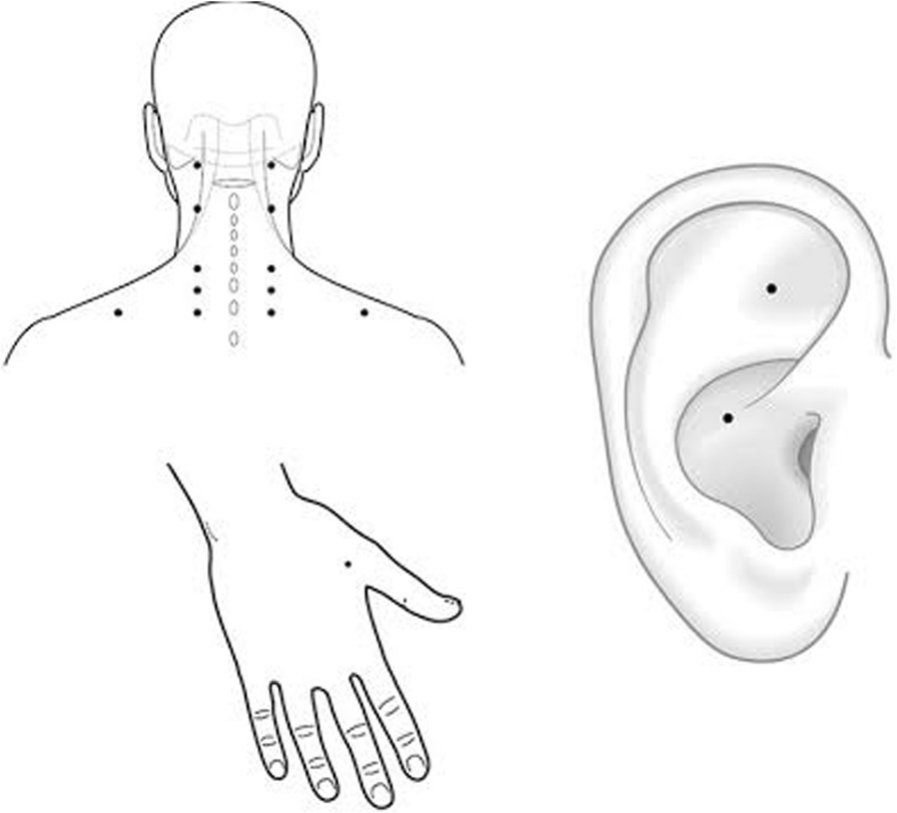
Anatomic needling points. Sixteen needles were inserted, from cephalad to caudal, bilaterally: superior and central area of the tip of the triangular fossa in junction of the superior and inferior crus of the antihelix (ear); helix root (ear); medial aspect of splenius capitis and semispinalis capitis muscles (C1-C2 level); lateral aspect of trapezius; semispinalis capitis muscle (C6-T1 level); levator scapulae muscles (C6-T1 level); abductor pollicis brevis and dorsal interossei muscles (hands).

We used acupuncture needles with guide tubes (Suzhou Huanqiu Acupuncture Medical Appliance Co. Ltd., 218, China) that were 40 mm in length and 0.25 mm in diameter. During the treatment, the patients sat comfortably, and their arms were supported. Needling areas were disinfected and disposable needles inserted at points previously selected as shown in Figure 2. The needles were placed over the anatomical areas as shown in Figure 2. From cephalad to caudal, needles were inserted bilaterally aiming to reach: medial aspect of splenius capitis and semispinalis capitis muscles at the level of C1-C2 vertebrae; lateral aspect of trapezius; semispinalis capitis muscle at the level of C6-T1 vertebrae. Also, at this same level, bilateral needling was performed at level of C6-T1 to reach the levator scapulae muscles. In the hand, the needles were inserted bilaterally to reach the abductor pollicis brevis and dorsal interossei muscles. In the ear, the needles were placed in two points: one in the superior and central area of the tip of the triangular fossa, between the junction of the superior crus and the inferior crus of the antihelix, and the other in the helix root, an area of innervation by the vagus nerve. A total of sixteen needles were used per subject per session. The acupuncture needles were connected to an electro-stimulator device (Model EL 608 NKL, Brusque, SC, Brazil) at an alternating frequency of 2 Hz and 10 Hz (2/10 Hz, 0.3 ms width) for 30 minutes. The intensity of the electrical therapy was adjusted to produce a gentle tapping sensation or up to the tolerance of the patient. The needles on the hand and ear were not electrically stimulated.

#### Sham

For the sham intervention, we used an electroacupuncture device (Cosmotron, Sao Paulo, Brazil), which was adjusted beforehand to prevent the current from passing through the electrodes. No needling was used at all. The electrical connection between the stimulator and the patient was broken at the output jack plug of the stimulator so that no current could pass to the patient. The patients were informed that this was a high-frequency, low-intensity stimulation and that they would most likely feel no sensation from it. The electrodes were placed in the same areas as the real stimulation. The nerve stimulation unit was left in front of the patient for 30 minutes. This positioning ensured that the flashing diode that simulated the electrical stimulus was both visible and audible. The patients were sitting comfortably, so they could see the device’s light sign.

### Instruments and assessments

All of the psychological tests used in this study have been validated for the Brazilian population [21,22]. One independent research examiner blinded to the group assignments was trained to administer the pain scales and to conduct the psychological tests. The baseline depressive symptoms of the patients were assessed using the Hamilton Depression Rating Scale. Catastrophic thoughts were assessed using the Brazilian Portuguese version of the Pain Catastrophizing Scale score [21]. The headache impact in daily life was assessed using the Short-Form Headache Impact Test (HIT-6) [22]. Demographic data and medical comorbidities were assessed using a standardized questionnaire. A systematic evaluation of potential complications of the technique, such as pneumothorax or bleeding, was also conducted.

### Outcomes

The primary outcomes were pain as assessed by the pain diaries (the maximum pain during the last 24 hours) and serum levels of BDNF. The secondary outcomes were the amount of analgesics used weekly throughout the treatment period.

The intensity of pain was measured by the 10-cm visual analog scale (VAS). The VAS scores ranged from no pain (zero) to the worst possible pain (10). The time of the worst pain during the last 24 hours was recorded daily in the patients’ diaries. They were asked to answer the following question: how intense was your worst pain during the last 24 hours? To improve patient compliance, an evaluator checked their pain records weekly.

The analgesic allowed to be used during the treatment period was acetaminophen 750 mg up to four times per day. In case it was not effective as a rescue analgesic, patients were allowed to use ibuprofen 200 mg a maximum of four times per day. If pain persisted, codeine 60 mg was also permitted. If codeine was ineffective, patients could use Dorflex® (Sanofi Aventis, Sao Paulo, Brazil; 35 mg Orfenadrine citrate combined with 300 mg Metamizol (Dypirone) and 50 mg caffeine). These medications could be used a maximum of four times a day. The analgesics used during the treatment period were monitored from diary entries recording analgesic intake, which were assessed in each treatment session. The total analgesic doses taken after starting treatment to the end were considered for the analysis.

The serum levels of BDNF were determined by Enzyme-Linked Immunosorbent Assay (ELISA) using a ChemiKine BDNF Sandwich ELISA Kit, CYT306, Chemicon/Millipore, Billerica, MA, USA. The lower detection limits of the kits are 7.8 pg/mL for BDNF. The data are expressed in ng/mL of blood serum. Blood samples were collected in plastic tubes with separator gel, centrifuged for 10 min at 4500 × g at 4°C, and serum was stored at −80°C for hormone assays. All Biological samples were collected early in the morning.

### Statistical analysis

Continuous and categorical variables were summarized using conventional descriptive statistics. Daily pain on the VAS was averaged per week and used to study pain behavior during the intervention periods. Normality was verified using the Shapiro-Wilk test. Variables were compared between the allocated sequence using independent samples *t*-tests for continuous variables and Chi-squared or Fisher’s exact tests for the categorical variables. Linear mixed models were used to compare outcomes within subjects during each intervention period (EA vs. Sham period) and between subjects according to the allocated phase (EA vs. Sham first). A carryover effect was defined as a significant difference in the pain score on the VAS between the two treatment sequence and was tested with the comparison provided by the linear mixed model. The mean differences between intervention periods according to sequence phase were calculated and presented with their respective standard error of the mean (SEM). The percentage of the change from baseline was calculated as 100-(outcome*100/baseline) and was presented with its respective 95% confidence interval (95%CI). The serum BDNF level was adjusted for severity of depressive symptoms (HDRS) and pain on the VAS using a multiple linear regression.

The standardized mean difference (SMD) was computed in terms of the ratio between the mean change and the pooled standard deviation. The SMD (also known as effect size) was interpreted as follows: small if lower than 0.20, moderate if between 0.50–0.60, and large if larger than 0.80 [21]. We considered all of the randomized patients as part of the analysis using the intention-to-treat method, with the last observation carried forward. A two-sided alpha level (type I error rate) of less than 0.05 was considered to be the statistical significance threshold. The data were analyzed using SSPS version 18.0 (SPSS, Chicago, IL).

## Results

Thirty-four patients were randomized (Figure 1). The clinical and demographic characteristics of the subjects according to the sequence allocation were comparable and are shown in Table 1. Seventeen patients were allocated to the phase receiving EA first and seventeen to the phase receiving sham first. Twenty-nine patients completed the study, and two participants in the EA first phase discontinued, one participant because of financial problems and the other participants because of personal problems. In the phase receiving sham first, three patients dropped out for different reasons: one because of lack of time to come to the center, another one because of dissatisfaction with the effect of the treatment and one who gave no justification. No adverse events were observed.

**Table 1:** **Characteristics of the study sample**

	**Sequence phase**		
	**Electroacupuncture first (n = 17)**	**Sham first (n = 17)**	***P***
Age (years)	39.11 (±10.5)	41.44 (±10.5)	0.36
Formal education (years of study)	14.44 (±3.9)	14.21 (±2.9)	0.85
Clinical comorbidity	4 (22%)	2 (12.5%)	0.66
Smoking	1 (5.5%)	0	1.00
Pain on the 10-cm VAS	6.02 (±1.5)	6.50 (±1.4)	0.28
HIT-6	63.00 (±6.4)	61.44 (±5.2)	0.44
HDRS	7.83 (±3.8)	6.69 (±2.8)	0.33
Psychiatric disease	6 (33.3%)	4 (25%)	0.59
B-PCS	32.17 (±13.5)	27.81 (±15.1)	0.38
Helplessness	14.17 (±6.9)	12.25 (±7.1)	0.43
Magnification	7.11 (±3.4)	5.69 (±3.8)	0.26
Rumination	10.78 (±3.7)	9.88 (±5,03)	0.55
Serum BDNF (ng/mL)	42.67 (±34.6)	43.2 (±21.4)	0.95
Daily use of analgesics (Yes/No)	14/3	15/2	0.66
Dorflex®	10	12	
NSAID	4	2	
Acetaminophen	4	2	0.81

Values are given as the mean (±SD) or as frequency (percentage of cases) (n = 34).

*Abbreviations:* *VAS* Visual analog scale, *HIT-6* headache impact test, *HDRS* Hamilton depression rating scale, *BP-PCS* Brazilian Portuguese pain catastrophizing scale, *B-PCS* pain catastrophizing scale validated for the Brazilian population, *BDNF* brain-derived neurotrophic factor, *NSAID* non-steroidal anti-inflammatory drug, Dorflex® (Sanofi Aventis, Sao Paulo, Brazil; 35 mg Orfenadrine citrate combined with 300 mg Metamizol (Dypirone) and 50 mg caffeine)).

**Figure 3:**
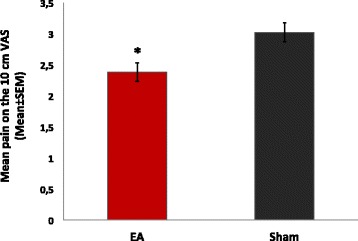
Mean pain by intervention period. Bars indicate the SEM. Electroacupuncture (EA) was superior to Sham in reducing pain. * P < 0.05.

**Figure 4:**
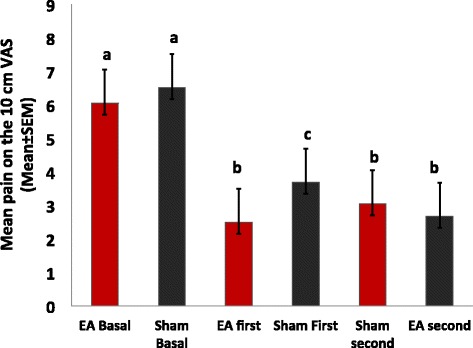
Mean pain according to intervention period and sequence phase. Bars indicate the SEM. Bars with the same letter are not significantly different from each other. Both interventions significantly reduced pain when compared to baseline. Electroacupuncture (EA) was superior to Sham in reducing pain.

EA was superior to sham reducing CTTH pain. During the EA period (irrespective of the phase), the mean (±SD) of the pain score on the VAS was 2.38 ± 1.77 (60.17% mean reduction from baseline) compared with 3.02 ± 2.49 during the sham period (38.49% mean reduction from baseline) (P = 0.005) (Figure 3). A significant difference was observed in the primary outcome (pain score on VAS) on the basis of treatment sequence (P < 0.05). Thus, a carryover effect was detected when the EA phase preceded the sham-treatment (Figure 4). The mean pain on the VAS during the EA period was not different than during the sham period (P = 0.29) in the EA-first phase. On the other hand, the mean pain differed between the intervention periods in the phase receiving the sham first (P = 0.003), with the VAS score lowering to 2.49 ± 1.79 during the EA period. Most of the patients (Eleven) had a reduction of 50% or more in the VAS during the EA period. In contrast, only five patients had that level of reduction in the sham-phase.

The serum BDNF was inversely correlated with the level of depressive symptoms and pain intensity, in the sense that more intense symptoms were associated with lower serum BDNF. Thus, the serum BDNF was adjusted for the HDRS and pain (weekly mean pain on the VAS) (r-squared = 0.07, standard β coefficient for the HDRS = −0.2, t = −3.87, standard β coefficient for the pain on the VAS = −0.14, t = −2.74, both *P* < 0.001). The mean of serum BDNF adjusted for depressive symptoms and pain was 30.01 ± 3.89 ng/mL in the phase receiving the EA first and 28.95 ± 3.45 ng/mL in the phase receiving sham first (Figure 4). At the end of the first EA period, the mean adjusted BDNF was 29.31 ± 3.24 ng/mL and at the end of the first sham period, it was 27.53 ± 2.94 ng/mL. The size of the effect of EA on the adjusted BDNF was moderate (Cohen’s *d* = 0.55).

EA significantly reduced the use of analgesics for CTTH. In the phase beginning with EA, the median number of analgesic doses used weekly (interquartile 25–75 range) was 2 (0–4) during the EA period and 1 (0–4) during the sham period (P = 0.04). In the phase beginning with sham, the median number of analgesic doses used weekly was 3 (0–7.25) during the sham period and 2 (0.5-4) during the EA period (P = 0.04).

## Discussion

**Figure 5:**
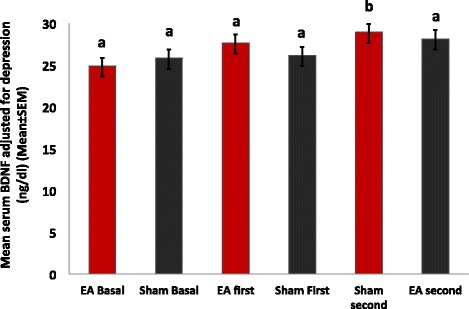
Serum BDNF adjusted for pain and depressive symptoms by intervention period and phase. Bars indicate the SEM. Bars with the same letter are not significantly different from each other. In those who received Electroacupuncture (EA) first, the serum BDNF elevation became significant after the Sham intervention, suggesting long-lasting effects of the EA.

In the present study, a relationship between pain and serum BDNF was demonstrated to be influenced by the timing of the EA intervention. In addition, our study demonstrated that the daily pain scores and number of analgesics used were significantly reduced in the phase receiving EA-first. This phenomenon was not observed in the phase starting with sham, confirming the carryover effect (Figure 4). After adjusting for depression and pain, serum BDNF changed in the opposite direction of the clinical outcomes, which suggests that, the elevation of serum BDNF levels after EA is inversely proportional to the perceived pain. In other words, the BDNF increased more after EA in those with greater analgesia (lower VAS) (Figure 5).

EA effects on both pain and BDNF were sustained beyond the real intervention periods. In addition to supporting the effects of EA described in other scenarios, such as neuropathic pain and depression [17,23,24], our findings provide a correlate between the clinical effect and a biological marker, the serum BDNF. The direction of the modulation on pain and BDNF persisted beyond the active intervention period; however, the underlying mechanisms of EA on these outcomes have not been fully elucidated yet. Nevertheless, our findings are in agreement with previous reports indicating that the increase in inhibitory activity and/or the decrease in excitatory synaptic activity may relate to BDNF [25]. In addition, the EA-first phase presented an analgesic effect of moderate magnitude, and the effect had a consistent magnitude with a previous meta-analysis report [26].

In our study, the EA analgesia was associated with the reinstatement of serum BDNF. This finding may be of relevance for clinicians and researchers because it reduces the gap between the apparent inconsistency of EA clinical effects reported in some meta-analysis [12], which has been questioned by experienced clinicians [27]. Furthermore, it extends additional neurobiological support for the sustained effect of EA on pain. This effect is biologically plausible considering the modulatory role of BDNF in the physiopathology of pain, as well as its involvement in neurogenesis and synapsis strengthening [28]. Such properties of the BDNF have been reported in other neuropathological conditions, such as brain ischemia [29] and after the partial dorsal rhizotomy [30]. In both conditions, the EA increased BDNF, which is similar to the effect observed after antidepressant administration in humans [31]. BDNF has been extensively studied in patients with major depression, and has been demonstrated that patients with the acute disorder have lower serum BDNF than when euthymic and than controls. Further, antidepressant treatment increases serum BDNF in those who respond to this pharmacological intervention [32]. Consistent with these reports, in our experiment we detected that serum BDNF was inversely correlated with depressive symptoms, maintaining the same direction of the observations in major depression: lower serum BDNF was associated with more intense depressive symptoms. Because of this, we adjusted for the effect of depressive symptoms on the serum BDNF in order to perceive better the effect of our intervention on the desired outcomes. After this adjustment, it was evident that the changes in BDNF were related to the EA intervention. To improve the understanding of the relationship between EA-induced analgesia and serum BDNF, different processes could be considered: BDNF can down-regulate the functions of NMDA receptors and inhibit the glutamate excitotoxicity [32], and it attenuates down-regulation of superoxide dismutase expression thereby decreasing free radical injuries [33] In addition, it regulates the expression of the calcium channel proteins on the cell membrane and may maintain the homeostasis of intracellular calcium [34]. In addition, BDNF regulates the expression of neuronal genes through upstream element-like transcriptional factors, including *c-fos* and *c-jun,* which are involved in the central sensitization process [35].

Even though a carryover effect was observed, these findings permitted us to highlight important points related to the acupuncture effect in the clinical setting. To the best of our knowledge, this is the first clinical study to extend the effect of EA in neuroplasticity markers indexed by the BDNF. In addition to the insights regarding neuroplasticity related to pain, our results also indicate that BDNF is a possible tool to improve the understanding of the disagreement between the clinical effect observed by clinicians and the failure of these techniques over placebo interventions in clinical trials. Although in general the carryover effect is interpreted as disadvantageous, in this study it was convenient, as it helped to reveal the serum BDNF as a possible marker to differentiate the effect of active treatment from those induced by the placebo.

In this study, the placebo effect reduced the pain 38.49%, a rate that is in agreement with previous studies that have reported a reduction in pain as high as 40% when using self-reported outcomes [36]. These findings are also in agreement with a meta-analysis that demonstrated that placebo effects are larger with more sustained pain and in the presence of hyperalgesia [37]. The placebo effect in pain studies may be explained by several patient-related reasons, such as: ***i)*** an established lower cognitive anchor point for pain and failure to sufficiently override prior beliefs when making reporting decisions; ***ii)*** over-weighting moments with lower pain experience when judging overall pain due to increased cognitive availability of experiences that match expectations; ***iii)*** desire to report what they believe the experimenter expectation is, in part because they believe this conforms to ‘correct’ or normative behavior; ***iv)*** desire to be consistent with prior behavior, which could include decreased reports of pain during prior treatment; and ***v)*** biasing of their reports towards what they would like to happen [38]. In fact, all these findings encourage us to promote the use markers of neuroplasticity, such as the BDNF, in future clinical studies to characterize the placebo effect.

Some considerations are relevant for a proper interpretation of our results. It is worth highlighting that in our trial a single experienced acupuncturist performed all the interventions. Although it could have reduced the intervention bias, it could also at the same time limit the ability of undertrained practitioners to reproduce the experiment, because slight variations in the technique could hypothetically induce different clinical responses. Nevertheless, because our research question focused on the neuroplasticity underlying the analgesic effect of electroacupuncture in chronic tension type headache, having an experienced acupuncturist providing an intervention of high quality reduced the risk of missing the signal of interest because of an inappropriately applied technique. Further, it is important to consider that because of the experimental design, even with an appropriate technique, it is impossible to determine whether the observed effect are purely due to the effect of the needle alone, electrical current alone, or due to both together.

The use of a crossover design in a small study population helped us to prevent overestimation of the benefits of the therapy being tested [39], making it likely that our results reflect a conservative assessment of the benefits of EA. The two-week washout period between study interventions was insufficient to prevent a carryover effect. The particular strength of this design is that the interventions under investigation were evaluated within the same patients and thus eliminates between-subject variability [40]. Given that patients act as their own controls, the analyses could be based on paired data (using paired tests) [41,42]. Also, because of this within-subject comparison, the *cross-over* design allows us to reduce the potential confounding effect that antidepressants and analgesics might have on the outcomes assessed. The carryover effect across intervention periods may distort the results obtained during the second intervention periods [43-45]. The number of patients who completed both treatment periods provided sufficient power (80%) to reach statistical significance (P < 0.05), despite 17.64% (3/17) of patients dropping out after the first intervention period and thus not receiving a second intervention. Although the outcome assessor, the care provider, and the patient were blinded (as recommended in the Delphi List for the quality of clinical trials), the attending acupuncturist was not because that was impossible. Finally, although several strategies were used to prevent patients and evaluator team from being unblinded, formal assessment for the awareness of the allocation (either active or placebo) was not performed. However, our objective surrogates are less prone to bias (i.e., serum BDNF and analgesic requirements) were consistent with the pain scores. Thus, unblinding is unlikely to have influenced the direction of our conclusions.

## Conclusion

In conclusion, these findings revealed that in patients with CTTH, the relationship between pain and serum BDNF was influenced by the timing of the EA intervention. The EA administered first increased the serum BDNF and facilitated a carryover effect that was associated with the pain scores and the number of analgesics used during the second intervention period. In addition, EA changed the serum BDNF in the opposite direction of depression and pain. Overall, these findings suggest that BDNF may be a serum marker of the neuroplasticity induced by EA.
